# Supplementary material for: Polymyxin B in Combination with Glycerol Monolaurate Exerts Synergistic Killing against Gram-Negative Pathogens
Source: Pathogens. 2022 Aug 2;11(8):874. doi: 10.3390/pathogens11080874 (PMC9413120; doi:10.3390/pathogens11080874)
Supplement: Supplementary file 1 [file pathogens-11-00874-s001.zip › pathogens-1800902-supplementary.pdf]

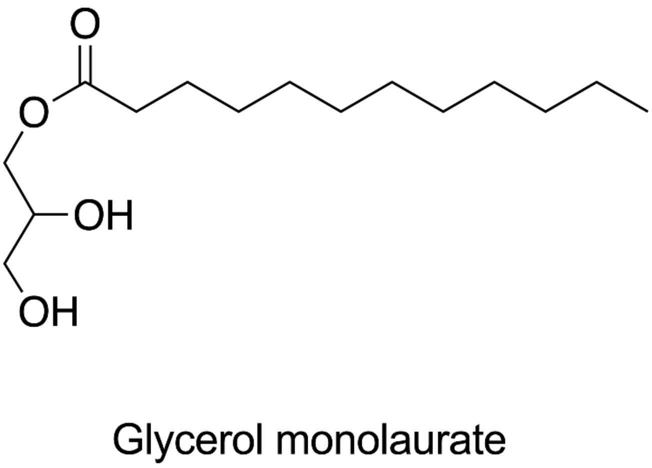

Figure S1. The structure of glycerol monolaurate.

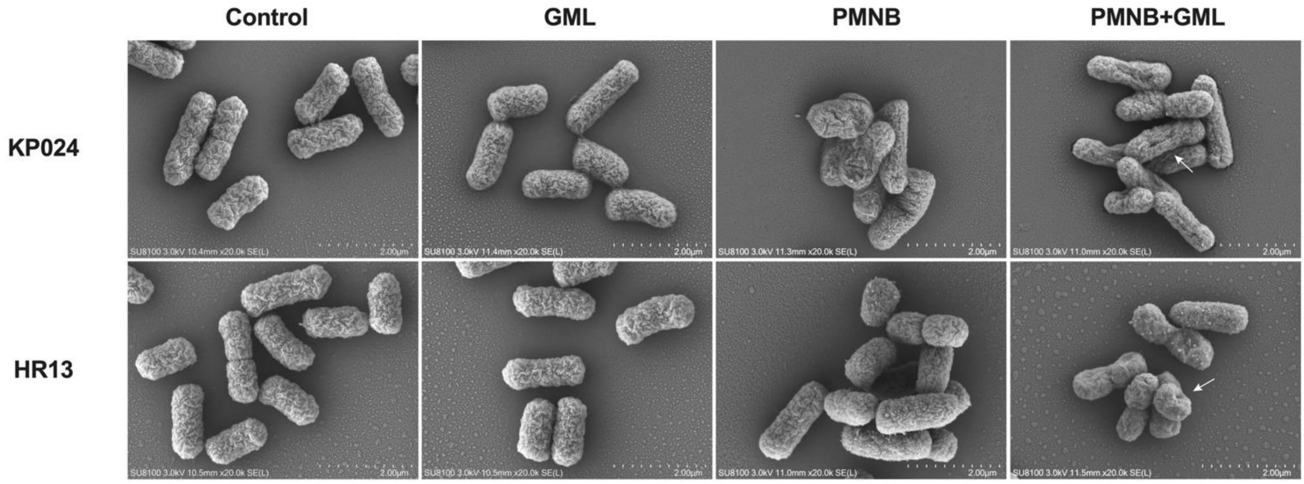

Figure S2. Cellular morphology of *K. pneumoniae* KP024 and *A. baumannii* HR13. SEM images of KP024 and HR13 after treatment with 4× MIC PMNB alone, GML (500 μg/mL) alone or in combination.

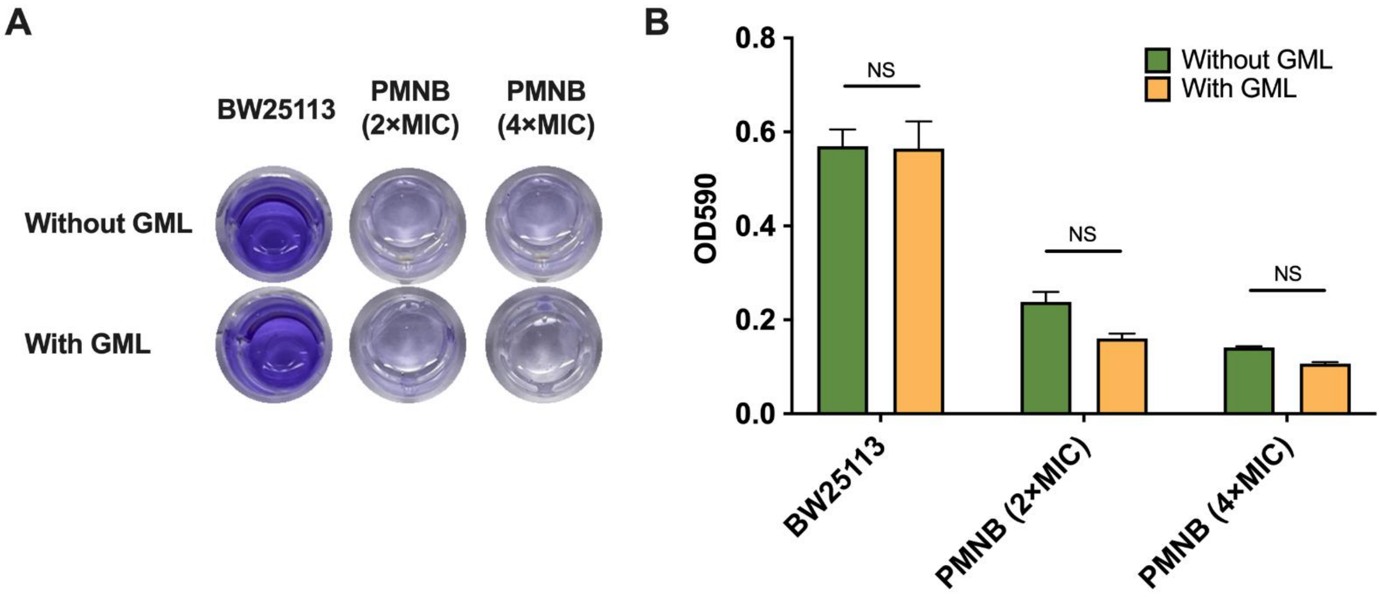

Figure S3. Impact of PMNB and GML on biofilm formation. **(A**) Crystal violet staining of *E. coli* BW25113 with different treatments. **(B)** Biomass quantification of biofilms by measurement of absorbance at 590 nm. PMNB, polymyxin B; GML, glycerol monolaurate.

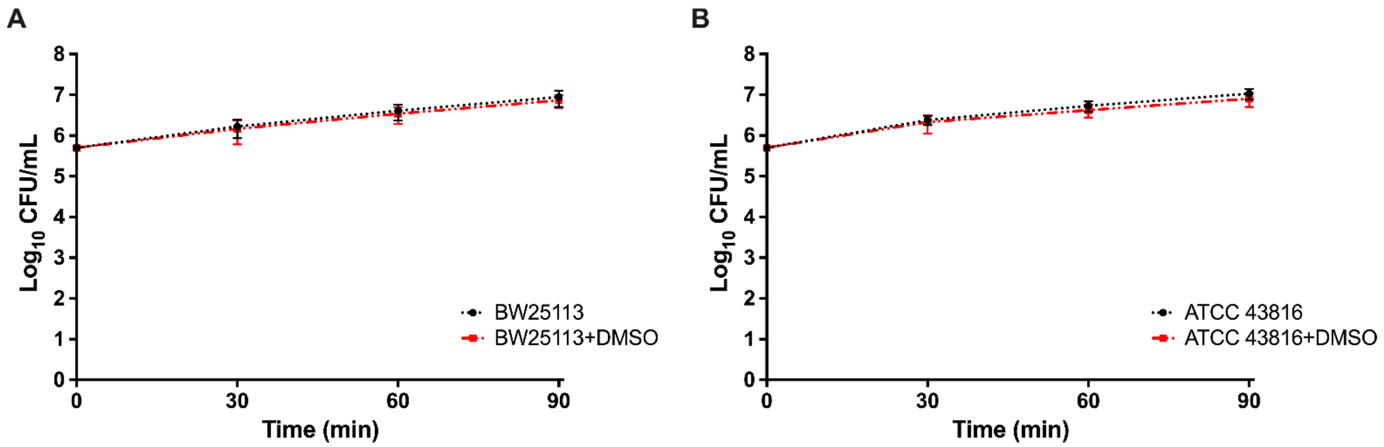

Figure S4. The effect of DMSO on **(A)** *E. coli* BW25113 and **(B)** *K. pneumoniae* ATCC 43816 growth kinetics. The addition of DMSO (5 %) alone did not alter bacterial growth *in vitro*. Results are representative of three independent experiments; error bars represent the standard errors of the means. P values (compared to untreated) are > 0.05.
